# Time dependency of thrombectomy for large artery atherosclerosis versus cardioembolic stroke subtypes: evidence from the ANGEL-ACT registry

**DOI:** 10.3389/fneur.2025.1574948

**Published:** 2025-05-27

**Authors:** Yue Yin, Hanlin Chen, Anxin Wang, Xiaoli Zhang, Miao Li, Ligang Song, Baixue Jia, Ning Ma, Dapeng Mo, Xuan Sun, Feng Gao, Yiming Deng, Zhongrong Miao

**Affiliations:** ^1^Department of Interventional Neuroradiology, Beijing Tiantan Hospital, Capital Medical University, Beijing, China; ^2^China National Clinical Research Center for Neurological Diseases, Beijing, China; ^3^Center of Stroke, Beijing Institute for Brain Disorders, Beijing, China; ^4^Department of Neurology, Beijing Tiantan Hospital, Capital Medical University, Beijing, China

**Keywords:** thrombectomy, time dependency, large artery atherosclerosis, cardioembolism, stroke subtypes

## Abstract

**Introduction:**

In this study, we investigated the differences in clinical outcomes following endovascular thrombectomy among ischemic stroke subtypes caused by large artery atherosclerosis (LAA) versus cardioembolism (CE) and the time-dependent nature of these clinical outcomes based on the stroke subtypes. Methods: Study participants were selected from the Endovascular Treatment Key Technique and Emergency Workflow Improvement of Acute Ischemic Stroke Registry to conduct a post-hoc analysis of a prospective, observational study. We included 1,046 patients, who had either LAA or CE stroke subtypes based on the Trial of Org 10172 in Acute Stroke Treatment criteria, drawn from the thrombectomy cohort. The association between clinical outcomes and time from stroke onset-to-recanalization time (ORT) was analyzed using a logistic regression model.

**Results:**

Overall, 545 (52.6%) and 491 (47.4%) patients were included in the LAA and CE groups, respectively. No significant difference was found in the 90-day clinical functional outcome between the LAA and CE patients when ORT was achieved within 240 min. Beyond 240 min, the rate of achieving a modified Rankin Scale score of 0–2 in patients with LAA was higher than that of patients with CE [48.17% versus 38.66%; odds ratio (OR) = 0.678, 95% confidence interval (CI) = 0.521–0.884, *p* = 0.0040], and after adjustment, the OR was 0.732 (95% CI: 0.537–0.998, *p* = 0.0486).

**Conclusion:**

In cases where the ORT exceeded 240 min, the clinical outcomes of patients with LAA were better than those of patients with CE, demonstrating a stronger time-dependency for achieving a favorable prognosis in patients with cardioembolic stroke.

## 1 Introduction

Mechanical thrombectomy (MT) has become the gold standard treatment for patients with acute large-vessel occlusion stroke, as demonstrated by multiple clinical studies (1–7). Guidelines recommend that patients with circulation occlusion undergo endovascular treatment (EVT) within 24 h of symptom onset, provided rigorous imaging screening is performed (8–10). Numerous studies have identified that surgical complications and clinical outcomes vary among patients with different stroke subtypes, suggesting that thrombectomy strategies should be tailored to the specific stroke subtype to optimize patient outcomes. Among patients with acute large vessel occlusion, cardioembolism (CE) is associated with an increased risk of hemorrhagic transformation within 24 h following EVT compared to large artery atherosclerosis (LAA) (11). For patients with vertebrobasilar occlusion stroke undergoing EVT, those with embolic stroke of undetermined origin have poorer outcomes and higher mortality rates compared to those with LAA or cardioembolic stroke (12). Additionally, in the cardioembolic group, the proportion of patients achieving a modified Rankin Scale (mRS) score of 0–2, indicative of good functional recovery, decreased as the onset-to-puncture time increased, a trend not observed in the LAA group (13).

Additionally, most current research focuses on the time window for patient selection but neglects the timing of surgical recanalization, including onset-to-door time, door-to-puncture time, puncture-to-recanalization time, and onset-to-recanalization time (ORT). Research on these times would be more beneficial for establishing better quality control metrics for thrombectomy. In this study, we aimed to explore the relationship between thrombectomy effectiveness and the ORT in patients with LAA and CE stroke.

## 2 Materials and methods

### 2.1 Study population

Data used in this study were obtained from the Endovascular Treatment Key Technique and Emergency Workflow Improvement of Acute Ischemic Stroke (ANGEL-ACT), a prospective nationwide registry comprising 2004 consecutive adult patients diagnosed with acute ischemic stroke (AIS) who underwent EVT across 111 hospitals in China between November 2017 and March 2019. Detailed methods of the registry, including inclusion and exclusion criteria as well as data collection standards, have been previously documented (14). The study protocol was approved by the ethics committee of each center, and all participants (or legal representatives) provided written informed consent. The study was conducted in accordance with the 1964 Declaration of Helsinki and its subsequent amendments.

**Figure 1:**
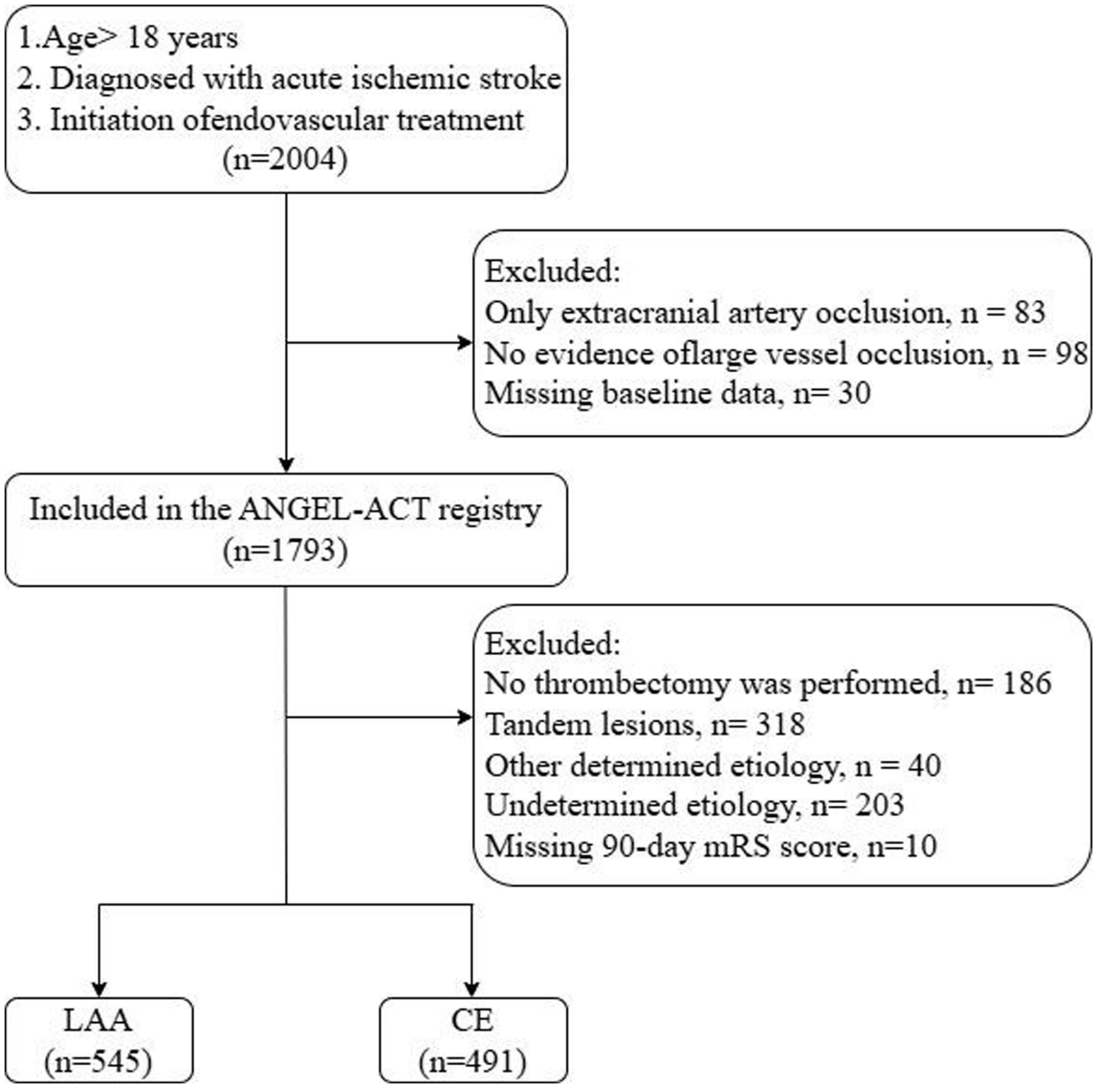
Patient selection flowchart. ANGEL-ACT, endovascular treatment key technique and emergency work flow improvement of acute ischemic stroke; LAA, large artery atherosclerosis; CE, cardioembolism.

This study included patients aged ≥18 years diagnosed with AIS who underwent initiation of EVT. Of the 2004 patients registered in the ANGEL-ACT database, 211 were excluded for the following reasons: 83 had isolated extracranial large-vessel occlusion, 98 had no evidence of large-vessel occlusion on digital subtraction angiography, and 30 had missing baseline data. Among the remaining 1793 patients, we further excluded those who did not undergo thrombectomy (*n* = 186), those with tandem lesions (*n* = 318), those with Trial of Org 10172 in Acute Stroke Treatment (TOAST) criteria for stroke of other determined etiology (*n* = 40), those with stroke of undetermined etiology (*n* = 203), and those whose 90-day mRS scores were missing (*n* = 10). Ultimately, 1,036 patients met the inclusion criteria for this analysis, comprising 545 with LAA and 491 with CE (Figure 1).

### 2.2 Data collection

Information regarding baseline demographic characteristics (age and sex), medical history (presence of hypertension, hyperlipidemia, or diabetes mellitus; prior stroke; mRS score ≥ 1 before stroke; use of GP IIb/IIIa receptor inhibitors; pretreatment with anti-platelets, intravenous thrombolysis, and anticoagulants), clinical features [systolic blood pressure (SBP), Alberta Stroke Program Early Computed Tomography Score (ASPECTS), National Institutes of Health Stroke Scale (NIHSS) score, onset-to-door time, door-to-puncture time, puncture-to-recanalization time, and ORT], location of the intracranial occlusion [anterior circulation included internal carotid artery, anterior cerebral artery (A1/A2), and middle cerebral artery (M1/M2); posterior circulation included vertebral, basilar, and posterior cerebral arteries (P1)], anesthesia type, and successful recanalization after the final attempt was documented.

### 2.3 EVT and stroke subtype

Prior to MT, intravenous thrombolysis was administered to eligible patients without contraindications. The choice between local and general anesthesia depended on the patient’s cooperation and condition. Following digital subtraction angiography, the neurointerventionist determined the optimal strategy and materials for the EVT. The surgical approach was determined based on the surgical situation and the personal experience of the neurointerventionist. To address *in situ* stenosis, several strategies were employed, including balloon expansion angioplasty alone (using Kalamazoo, MI, USA, Gateway, Stryker; or Neuro-RX SINOMED, Tianjin, China), balloon-mounted stents alone (such as Apollo, MicroPort, Shanghai, China), or a combination of balloon-mounted and self-expanding stents (Enterprise, Codman & Shurtleff Inc., Miami, FL, USA; Wingspan or EZ, Stryker, Kalamazoo, MI, USA; or Solitaire AB, Medtronic, Minneapolis, Minnesota, USA), following balloon expansion.

Stroke subtype classification was performed by two separate neurologists or onsite investigators using the 1993 version of the TOAST criteria (15). Prior to patient enrollment, all evaluators underwent training by committee-assigned stroke specialists and were provided with a manual containing detailed descriptions of the TOAST subtyping system and operational guidelines for determining the etiological subtype. Diagnosis of CE stroke typically requires identification of at least one cardiac source of embolism (via electrocardiogram or echocardiogram), characterized by clinical symptoms and brain imaging findings demonstrating occlusion of a major cerebral or branch cortical artery. Using a standardized diagnostic process, trained evaluators reviewed the patients’ clinical history, imaging findings, and laboratory features and categorized them into LAA or CE stroke subtypes according to the TOAST system.

### 2.4 Outcome measurement

Experienced investigators meticulously recorded all data. We considered a favorable functional recovery outcome at 90 days post-procedure (defined as a 90-day mRS score of 0–2) as the efficacy endpoint. We also recorded the onset-to-door time, door-to-puncture time, puncture-to-recanalization time, and ORT.

### 2.5 Statistical analysis

Data are presented as the median (interquartile range) or frequency (percentage). A comparison of baseline characteristics between patients with LAA and those with CE was performed using the Kruskal–Wallis test or chi-square test, as appropriate. A multivariate logistic regression model was used to examine the association between the stroke subtypes (LAA vs. CE) and ORT. Variables including age; sex; presence of hypertension, diabetes mellitus, or hyperlipidemia; SBP; baseline NIHSS score; ASPECTS; and pretreatment with antiplatelet agents (aspirin and clopidogrel) were adjusted for in the analysis. The adjusted odds ratio (OR) with 95% confidence intervals (CIs) was calculated to measure the strength of the association.

**Figure 2:**
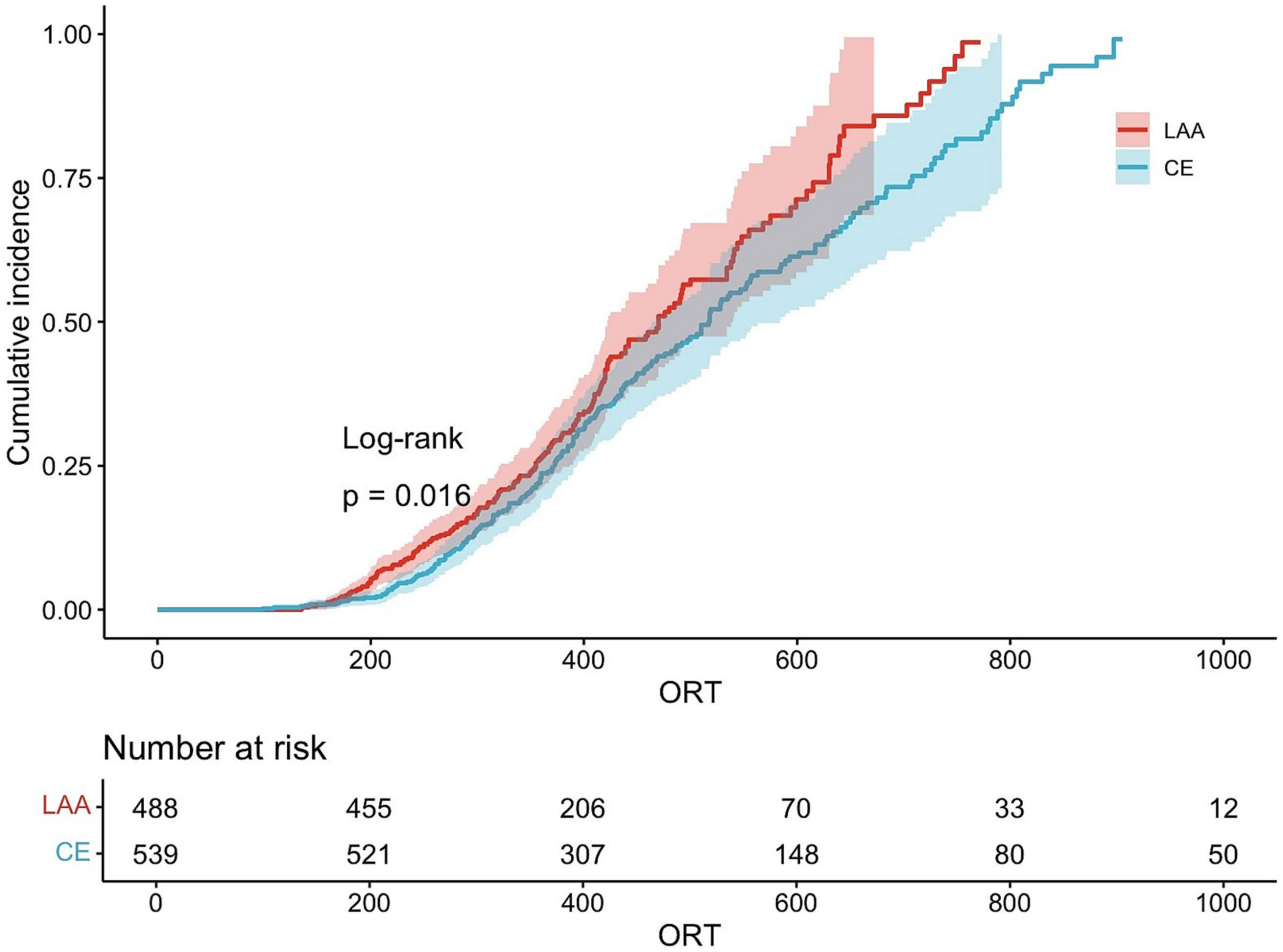
Cumulative percentage of good prognoses. LAA, large artery atherosclerosis; CE, cardioembolism.

The cumulative percentage of good prognoses was used to determine the point at which the prognosis in the LAA group surpassed that in the CE group (Figure 2).

According to the statistical data, we analyzed the proportion of mRS scores of 0–2 across various time segments and identified 240 min as the optimal cutoff. The correlation between good functional outcomes and ORT in the two groups was analyzed separately for cases occurring before and after 240 min.

Mediation models were used to examine whether the relationship between stroke subtype and ORT was mediated by the following factors: NIHSS score, site of occlusion, prior use of anticoagulants, type of thrombectomy (stent retriever alone or stent retriever plus aspiration), number of thrombectomy passes, and emergency angioplasty/stenting. The detailed methods of mediation analysis can be found in Supplementary Material. All statistical analyses were conducted using SAS software (version 9.4; SAS Institute, Inc., Cary, NC, USA). A two-sided *p*-value <0.05 was considered statistically significant.

## 3 Results

### 3.1 Baseline characteristics of patients with LAA versus CE

Of the 1,036 patients included in this study, 545 with LAA and 491 with CE were compared for baseline characteristics. As shown in Table 1, several baseline variables differed significantly between the two groups. Compared with the LAA group, the CE group exhibited the following characteristics: (1) a higher mean age (69 vs. 63 years); (2) a higher median NIHSS score (17 vs. 16); (3) a higher median ASPECTS (10 vs. 8); (4) a significantly larger proportion of women (52.55% vs. 23.12%); (5) a greater percentage of anterior circulation strokes (87.78% vs. 70.28%); and (6) a greater percentage of pretreatment with anticoagulants (8.35% vs. 1.47%). Meanwhile, the CE group had a smaller proportion of patients with hypertension (54.38% vs. 60.92%), diabetes mellitus (14.66% vs. 20.18%), and those who received antiplatelet agents before the procedure (12.83% vs. 17.25%). The CE group used GP IIb/IIIa receptor inhibitors less frequently during the procedure (35.23% vs. 72.11%). However, the CE group experienced shorter onset-to-door times (135 vs. 170 min), shorter door-to-puncture times (114 vs. 127 min), and shorter ORTs (367 vs. 432 min) than the LAA group. No significant difference was found between the two groups regarding the presence of hyperlipidemia, prior stroke, mRS scores ≥ 1 before stroke, initiation of intravenous thrombolysis pretreatment, type of anesthesia, puncture-to-recanalization time, or complete recanalization after the final attempt (Table 1).

**Table 1:** Baseline characteristics of the patients with LAA and CE stroke.

	LAA (*n* = 545)	CE (*n* = 491)	*p* value
Age, y, median [IQR]	63 [54–71]	69 [62–77]	<0.0001
Hypertension, *n* (%)	332 (60.92)	267 (54.38)	0.0333
Male, *n* (%)	419 (76.88)	233 (47.45)	<0.0001
Hyperlipidemia, *n* (%)	62 (11.38)	41 (8.35)	0.1041
Diabetes Mellitus, *n* (%)	110 (20.18)	72 (14.66)	0.0197
Prior stroke, *n* (%)	103 (18.90)	100 (20.37)	0.5524
mRS ≥ 1 before stroke, *n* (%)	65 (11.93)	64 (13.03)	0.5988
SBP, mmHg, median [IQR]	149 [133–165]	145 [130–160]	0.0040
Baseline NIHSS, median [IQR]	16 [11–21]	17 [13–21]	0.0013
ASPECTS, median [IQR]*	8 [7–10]	10 [7–10]	<0.0001
Occlusion site, *n* (%)			<0.0001
Anterior circulation	383 (70.28)	431 (87.78)	
Posterior circulation	162 (29.72)	60 (12.22)	
Anesthesia type, *n* (%)			0.6245
General anesthesia	208 (38.17)	176 (35.85)	
Local anesthesia only	238 (43.67)	229 (46.64)	
Local anesthesia plus sedation	99 (18.17)	86 (17.52)	
Pretreatment with anti-platelets, *n* (%)	94 (17.25)	63 (12.83)	0.0477
Pretreatment with IVT, *n* (%)	145 (26.61)	144 (29.33)	0.3293
Pretreatment with anticoagulants, *n* (%)	8 (1.47)	41 (8.35)	<0.0001
Complete recanalization after the final attempt†	376 (68.99)	364 (74.13)	0.0673
GP IIb/IIIa receptor inhibitor, *n* (%)	393 (72.11)	173 (35.23)	<0.0001
Onset-to-Door time, min, median [IQR]	170 [78–330]	135 [61–260]	0.0058
Door to Puncture time, min, median [IQR]	127 [90–191]	114 [74–159]	<0.0001
Puncture-to -recanalization time, min, median [IQR]	83 [50–127]	80 [50–117]	0.5803
Onset-to-recanalization time (ORT), min, median [IQR]	432 [315–628]	367 [277.5–492.5]	<0.0001

LAA, large artery atherosclerosis; CE, cardioembolism; IQR, interquartile range; mRS, modified rankin scale; SBP, systolic blood pressure; mTICI, modified thrombolysis in cerebral infarction; NIHSS, national institutes of health stroke scale; ASPECTS, alberta stroke program early CT score; pc-ASPECTS, posterior circulation alberta stroke program early CT score; IVT, intravenous thrombolysis.

*ASPECTS for anterior circulation stroke and pc-ASPECTS for posterior circulation stroke.

†Defined as an mTICI score of 3.

### 3.2 Distribution of mRS scores across ORT categories and stroke subtypes

Among all enrolled participants, patients with LAA stroke had a higher percentage of favorable functional outcomes (mRS score of 0–2) than those with CE stroke. In the subgroup with an ORT < 240 min, 54.72% of patients with LAA stroke and 59.72% of patients with CE stroke achieved mRS scores of 0–2. In the subgroup with an ORT ≥ 240 min, 48.17% of patients with LAA stroke and 38.66% of patients with CE stroke achieved mRS scores of 0–2 (Table 2).

**Table 2:** mRS scores of 0–2 for different ORT categories in patients with LAA and CE stroke.

Variable	LAA		CE	
	mRS score 0–2	mRS score 0–6	mRS score 0–2	mRS score 0–6
Total	266 (48.81%)	545	205 (41.75%)	491
ORT = 0–240 min	29 (54.72%)	53	43 (59.72%)	72
ORT> = 240 min	237 (48.17%)	492	162 (38.66%)	419
ORT = 241–480 min	138 (50.18%)	275	106 (37.19%)	285
ORT = 481–720 min	47 (39.17%)	120	30 (35.71%)	84
ORT> = 721 min	52 (53.61%)	97	26 (52.00%)	50

### 3.3 Logistic regression analysis of ORTs and mRS scores of 0–2 in patients with LAA and CE

Table 3 presents the results of the single and multifactor analyses examining the association between the ORTs and favorable functional outcomes (as indicated by mRS scores of 0–2). For cases with an ORT < 240 min, there was no statistically significant difference in the rate of favorable functional outcomes between the LAA and CE groups (OR: 1.227; 95% CI: 0.599–2.514; *p* = 0.5759). Similar results were observed in the adjusted models, with *p* > 0.05. After adjusting for age, sex, high blood pressure, presence of diabetes mellitus, presence of hyperlipidemia, SBP, baseline NIHSS score, ASPECTS score, pretreatment with anti-platelets, and pretreatment with anticoagulants in the adjusted model, the OR was 1.633 (95% CI: 0.700–3.810, *p* = 0.2564).

**Table 3:** Odds ratio of mRS scores distribution in LAA versus CE stroke.

mRS scores of 0–2		OR	*P*-value	OR (Adjusted MODEL)	*P*-value
LAA vs. CE	ORT<240 min	1.227 (0.599–2.514)	0.5759	1.633 (0.700–3.810)	0.2564
	ORT≥240 min	0.678 (0.521–0.884)	0.0040*	0.732 (0.537–0.998)	0.0486*

Adjusted Model: Logistic regression adjusted for age, gender, high blood pressure, presence of diabetes mellitus, presence of hyperlipidemia, systolic blood pressure, baseline NIHSS score, ASPECTS score, pretreatment with anti-platelets, and pretreatment with anticoagulants.

*Statistically significant.

For cases with an ORT ≥ 240 min, there was a statistically significant difference in favorable functional outcomes between the LAA and CE groups. The unadjusted OR was 0.678 (95% CI: 0.521–0.884; *p* = 0.0040), indicating that CE stroke is a risk factor compared to LAA stroke. Similar results are obtained for the adjusted model. After adjustment, the OR was 0.732 (95% CI: 0.537–0.998, *p* = 0.0486).

## 4 Discussion

To the best of our knowledge, this is the first study to investigate the association between the ORT and favorable clinical functional recovery (defined as mRS scores of 0–2) in patients with CE and LAA stroke subtypes. The study indicated no significant difference in clinical outcomes between patients with LAA and CE stroke within 240 min of stroke onset. However, beyond 240 min, the clinical outcomes of patients with LAA stroke were better than those of patients with CE stroke, demonstrating a stronger time dependency for favorable prognosis in patients with CE stroke. A possible reason for this is that patients with LAA stroke tend to have better collateral recruitment due to the chronic process of ischemic preconditioning (16). Researchers have found that patients with stroke due to cervical carotid atherosclerosis had better cerebral collateral circulation and slightly better median mRS scores at 90 days compared to those with CE stroke (17).

The findings suggest that CE stroke patients have a stronger time-dependent aspect. While early recanalization remains critical for both subtypes, CE strokes exhibit a stronger decline in favorable outcomes with delayed reperfusion, likely due to shortage of collateral circulation. Therefore, we recommend shortening the time from symptom onset to recanalization in patients with CE. Understanding the implications of these findings can significantly impact various aspects of stroke management.

Firstly, CE stroke was associated with a higher risk of any hemorrhagic transformation compared to LAA stroke (11). One possible explanation is that the difference in thrombus composition between stroke subtypes may account for the higher number of thrombectomy passes associated with CE stroke. These findings underscore the importance of accurately identifying the stroke subtype.

Secondly, given the time-sensitive nature of CE stroke outcomes, the choice of devices that can facilitate quicker and more efficient reperfusion is crucial to optimize the chances of favorable outcomes within the critical time window. Moreover, We found that the CE group experienced shorter onset-to-door times, shorter door-to-puncture times, and shorter ORTs than the LAA group. We hypothesize that CE patients presented with more severe symptoms (e.g., higher NIHSS due to abrupt vessel occlusion), prompting faster triage (18).

Finally, establishing stringent quality control standards for CE patients is essential for ensuring optimal clinical outcomes. Stringent medical quality control is crucial for a country like China with an uneven healthcare distribution.

This study has some limitations. Firstly, this was not a randomized study, and thus can only partly illustrate the issue. This study is a real-world investigation with a large sample size. However, due to the relatively early timeframe, the efficiency of thrombectomy was not very high, possibly because of insufficient thrombectomy experience and equipment. During the study period, researchers found that a subset of the population underwent direct aspiration as the primary thrombectomy approach, which was linked to lower rates of successful recanalization with the initial device, indicating the need for employing additional rescue treatments, and a higher risk of intracranial hemorrhage compared to using a stent retriever as the primary thrombectomy approach (19). Secondly, there is a high portion of patients with intracranial athero-occlusive disease in China, which decreases the external validity or generalizability of the findings.

In conclusion, this study suggests that patients with LAA stroke had better clinical outcomes compared to those with CE stroke when the ORT ≥ 240 min, demonstrating a stronger time-dependency for favorable prognoses in CE stroke. Further research is necessary to elucidate the factors mediating the relationship between stroke subtypes and the ORT and to inform the development of clinical management strategies.

## Data Availability

The original contributions presented in the study are included in the article/Supplementary material, further inquiries can be directed to the corresponding author/s.
