# Supplementary material for: Time dependency of thrombectomy for large artery atherosclerosis versus cardioembolic stroke subtypes: evidence from the ANGEL-ACT registry
Source: Front Neurol. 2025 May 27;16:1574948. doi: 10.3389/fneur.2025.1574948 (PMC12150852; doi:10.3389/fneur.2025.1574948)
Supplement: Supplementary file 1 [file Table_1.docx]

Continuous Mediation Analysis of the LAA stroke subtype & OTR time

Model 1: Adjusted for age

Model 2: Model 1+high blood pressure, presence of diabetes mellitus, presence of hyperlipidemia, systolic blood pressure, baseline NIHSS score, final ASPECTS score, aspirin use, and clopidogrel use

Unadjusted mediation effect: P=0.1377

Model1 mediation effect: P=0.2368

Model2 mediation effect: P=0.2623

Unadjusted mediation analysis

NIE=0.003058

OTR

C2=-0.00015

C1=-20.9017

NDE=-0.04263

TOTAL=-0.03957

LAA

MRS0-2

Continuous Mediation Analysis of the LAA stroke subtype & OTR time

Model 1: Adjusted for age

Model 2: Model 1+high blood pressure, presence of diabetes mellitus, presence of hyperlipidemia, systolic blood pressure, baseline NIHSS score, final ASPECTS score, aspirin use, and clopidogrel use

Unadjusted mediation effect: P= 0.1224

Model1 mediation effect: P= 0.2461

Model2 mediation effect: P= 0.2524

Unadjusted mediation analysis:

NIE= 0.003502

OTR

C2= -0.1552

C1= -0.01855

NDE= -0.04307

TOTAL= -0.02013

LAA

MRS0-2
